# Left Ventricular Thrombus After Acute Decompensated Heart Failure in the Setting of Ischemic Cardiomyopathy

**DOI:** 10.7759/cureus.4537

**Published:** 2019-04-24

**Authors:** Mohan Satish, Naveen Vukka, Dinesh Apala, Toufik Mahfood Haddad, Jaya Gupta

**Affiliations:** 1 Internal Medicine, Creighton University School of Medicine, Omaha, USA; 2 Cardiology, Creighton University School of Medicine, Omaha, USA

**Keywords:** lv thrombus, heart failure, ischemic cardiomyopathy

## Abstract

A 70-year-old male with a medical history significant for long-standing ischemic cardiomyopathy (ICM) and heart failure with reduced ejection fraction (HFrEF) was admitted to the hospital with shortness of breath (SOB) five days after an acute heart failure (HF) exacerbation. He had non-radiating chest pressure now at rest, but without evidence of an acute coronary syndrome (ACS). Diagnostic work-up on readmission included a transthoracic echocardiogram (TTE), which revealed worsening left ventricular (LV) systolic dysfunction with new wall motion abnormalities and an incidental echo density in the LV apex, suggestive of an LV thrombus. These findings were unseen on imaging 20 months prior. The patient was initiated on warfarin to be maintained for three months, and discharged in stable condition after optimization of his anginal symptoms. Cardiac catheterization was not attempted secondary to the patient’s chronic kidney disease (CKD). The incidental finding of an LV thrombus occurred despite compliance with guideline-directed medical therapy of HFrEF and ICM, including adjunctive use of clopidogrel. With the poor survival associated with thromboembolism, the prevention, risk stratification and appropriate therapeutic approach to LV thrombus are poorly delineated in patients with HFrEF in sinus rhythm. Currently, the screening guidelines for the identification of LV thrombus in patients with HFrEF are also unknown. Given mixed evidence regarding prophylactic anticoagulation, we present this case of an incidental LV thrombus found during an episode of acute decompensated HF in the setting of long-standing ICM to emphasize the need to suspect LV thrombus formation after such presentations with closer follow-up for prompt detection and timely treatment.

## Introduction

A left ventricular (LV) thrombus is a complication of severe LV systolic dysfunction, most notably secondary to anterior myocardial infarction (MI), chronic heart failure (CHF) and dilated cardiomyopathy [1-2]. The pathophysiology of LV thrombus formation relates to factors within these etiologies associated with Virchow’s triad - endothelial injury, hypercoagulability, and stasis of blood flow [3]. In HF with reduced ejection fraction (HFrEF), a hypercoagulable state is noted with the increased incidence of LV thrombus and a higher risk of thromboembolism [4]. In light of these findings, prophylactic systemic anticoagulation has been considered [5]. However, in HFrEF (EF ≤35%) patients in sinus rhythm, prophylactic systemic anticoagulation is not supported relative to the increased risk of bleeding [6-9]. Therefore, timely identification of an LV thrombus in patients with HFrEF is necessary to avoid delays in treatment. We present a patient with HFrEF and ischemic cardiomyopathy (ICM) that was identified to have LV thrombus during an encounter with acute HF exacerbation.

## Case presentation

**Figure 1:**
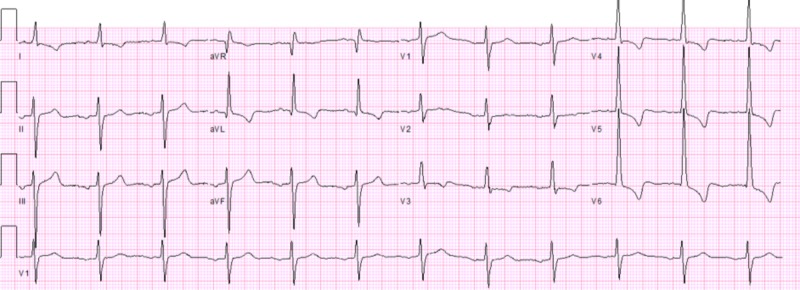
Electrocardiogram findings during first admission

A 70-year-old Caucasian male with a medical history significant for long-standing ICM with HFrEF (EF = 40% w/ grade I diastolic dysfunction), chronic kidney disease (CKD) stage III B, and hypertension presented to the emergency room (ER) with complaints of acute onset non-radiating exertional chest pressure, orthopnea, and shortness of breath (SOB). Eight months prior, the patient had presented with similar symptoms and was treated medically for an acute exacerbation of his HF (New York Heart Association Class III). Physical examination revealed 2+ bilateral pitting pedal edema and diffuse crackles on lung auscultation. Review of outpatient medications revealed optimized HFrEF guideline-directed medical therapy and the patient endorsed compliance. Initial workup was significant for notable elevations in N-terminal-pro hormone B-type natriuretic peptide (NT-proBNP) (to >4000 pg/mL) and low suspicion of the acute coronary syndrome (ACS) from unchanged electrocardiogram (ECG) findings (Figure 1) and stable cardiac enzyme levels. Subsequent chest x-ray (CXR) was significant for signs of pulmonary edema including prominent cardiomegaly. The patient was admitted for acute exacerbation of HF and underwent successful intravenous (IV) diuresis, and discharged two days after admission. He stated the resolution of symptoms at the time of discharge. Echocardiography was considered appropriate for outpatient follow-up. 

**Figure 2:**
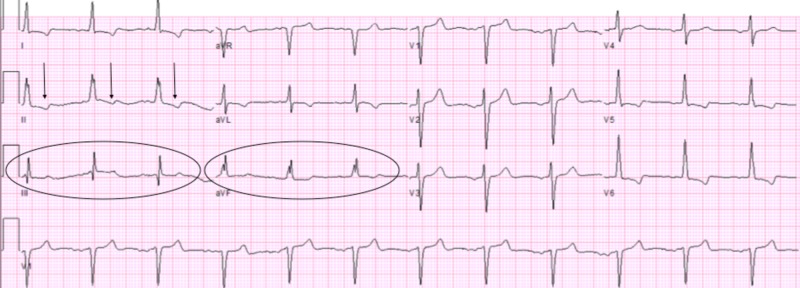
Electrocardiogram findings during second admission T-wave inversions (*arrows*), depressed T-wave amplitudes (*circled inferior leads*)

**Figure 3:**
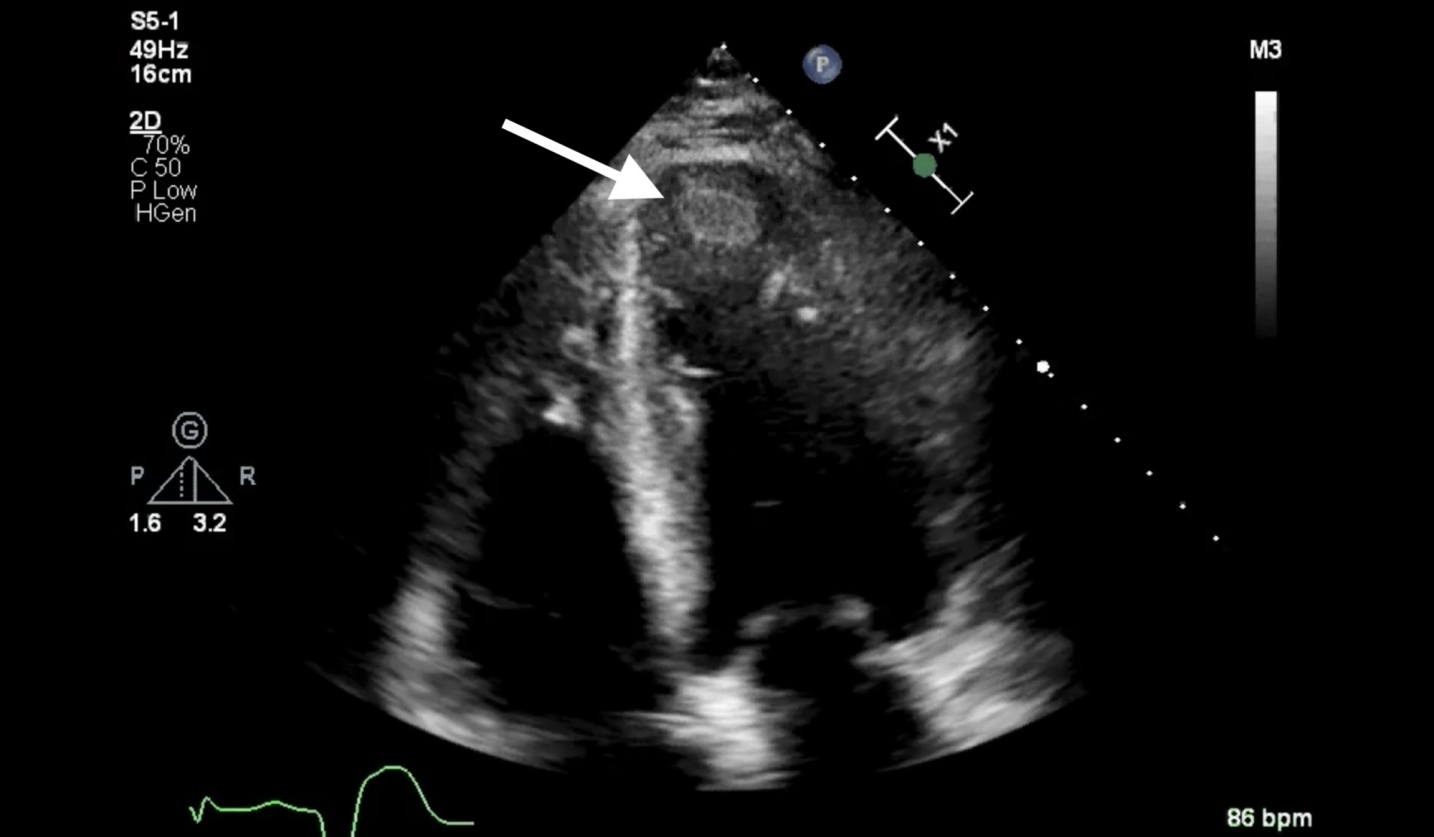
Echocardiographic findings of left ventricular apical thrombus

However, the patient returned to the ER five days after initial discharge with complaints of SOB and similar non-radiating chest pressure now present at rest. He was admitted for observation given persistent chest pain in the setting of significant cardiac history. Diagnostic workup included ECG, which was significant for new T-wave changes in the inferior leads concerning for possible ischemia (Figure 2) and transthoracic echocardiogram (TTE), which was significant for further reduction in LV systolic function with EF noted to be 30-35%, with an akinetic apex. Additionally, an immobile 1.6 cm x 1.4 cm echo density was found in the LV apex suggestive of an LV thrombus (Figure 3). Compared to the patient's prior cardiac imaging, TTE performed 20 months prior showed mild global LV hypokinesis with EF 35-40% without evidence of LV thrombus. Cardiology was consulted and recommended anticoagulation with warfarin for three months as well as titration of a long-acting nitrate for better control of anginal symptoms. The patient was discharged in a stable condition after medication optimization.

## Discussion

The presence of LV thrombus secondary to severe LV systolic dysfunction (<35%) has been reported at a rate of around 20%, with ICM alone found to be an independent predictor of thrombus formation and thromboembolism [10]. Using two-dimensional speckle-tracking echocardiography (2D-STE), patients with HFrEF with LV thrombus formation have been associated with reduced longitudinal strain in the inferior and apical regions, and in the territory of the left anterior descending compared to HFrEF patients without LV thrombus formation [11]. Current American College of Cardiology (ACC)/ American Heart Association (AHA) Heart Failure guidelines consider anticoagulation without prior thromboembolic event or cardioembolic source to be a class III recommendation in patients with HFrEF who are in sinus rhythm. Given higher rates of mortality in patients with HFrEF suffering a thromboembolic event, timely identification of an LV thrombus is crucial for untreated patients in sinus rhythm without known thromboembolic risk [10]. 

Currently, the screening guidelines for identification of LV thrombus in patients with HFrEF are unknown. After AMI, it has been suggested that TTE should be performed in patients at high risk for apical LV thrombus (i.e. anterior MI or delayed reperfusion) within 24 hours of admission and consideration of follow up imaging within one to three months [11]. Unlike AMI, however, risk stratification for thromboembolism in patients with HFrEF in sinus rhythm is not well understood, and may also be temporally related with a higher risk of thrombus occurring more proximal to MI [12]. Such risk stratification would better address the guidelines for LV thrombus screening by means of TTE or cardiac magnetic resonance imaging (MRI), which may better inform further studies regarding the improved risk-benefit profile of novel oral anticoagulants (NOACs) in this setting [13]. Underscoring the need for such an outlook in HFrEF, we report a case where the incidental finding of an LV thrombus highlighted its vague clinical presentation. Additionally, this case also identifies the unclear guidelines to prevent an LV thrombus in the setting of this patient’s HF that was medically optimized and included adjunctive use of clopidogrel for his ICM.

In the case described, the patient had long-standing ICM of nearly 13 years with associated HFrEF that had been both optimized by revision of his three-vessel coronary artery bypass (CABG) five years prior and medical management. Additionally, the patient’s last TTE, 20 months prior to his presentation, failed to show significant changes in pump function and major wall motion abnormalities since his revision. It is likely that his HFrEF, perhaps in relation to worsening ICM, became refractory to optimized medical management, noted by his initial presentation of acute decompensated HF eight months prior to identifying his LV thrombus. Considering worsening ICM, the patient’s advanced CKD prohibited cardiac catheterization to characterize this further. In this case, repeated cardiac imaging would have provided temporal information regarding the thrombus formation in the last 20 months. Given that ICM is an independent risk factor for LV thrombus formation, in addition to severe LV systolic dysfunction alone with sinus rhythm, it may be that acute decompensated HF, particularly refractory HF requires closer follow-up that is guided temporally. After compensation of the patient’s exacerbation occurring eight months prior, cardiac imaging would not only have been helpful to identify an LV thrombus, it would have assessed worsening and permanent wall motion abnormalities (apical akinesis or severe global hypokinesis) associated with increased risk for its formation. In the patient’s subsequent course described, these were found with the incidental identification of the LV thrombus. While the patient was scheduled for outpatient follow-up for echocardiography after the first admission detailed, the detection of his LV thrombus would remain incidental to the concern of worsening ventricular function. Additionally, only a 42% utilization rate was noted by Goyfman et al. with follow-up echocardiogram performed during an index admission for acute decompensated HF in the setting of medication noncompliance where such risk is higher [14]. Irrespective of prophylactic anticoagulation, the unclear risk stratification for thrombus formation in patients with HFrEF may be addressed by evaluating specific cardiac imaging findings (i.e. wall motion abnormalities) and the independent risk of ICM in confirmatory randomized studies. Better risk stratification will also help to address the need for individualized approaches to prevent thromboembolism in patients with HFrEF.

## Conclusions

With the poor survival associated with thromboembolism, the prevention, risk stratification and appropriate therapeutic approach to LV thrombus are poorly delineated in patients with HFrEF in sinus rhythm. We present a case of an incidental LV thrombus found after acute decompensated HF in the setting of long-standing ICM and HFrEF to emphasize the need to consider LV thrombus formation in such patients with close follow-up. Likewise, without evidence for prophylactic anticoagulation, identifying patients at highest risk of LV thrombus formation will refine guidelines for cardiac imaging to increase the prompt detection and treatment of these patients to thereby prevent fatal thromboembolism.
